# Clinical and economic impact of pharmacist interventions on sampled outpatient prescriptions in a Chinese teaching hospital

**DOI:** 10.1186/s12913-018-3306-4

**Published:** 2018-07-04

**Authors:** Zhiwei Bao, Chunmei Ji, Jing Hu, Can Luo, Wentong Fang

**Affiliations:** 1 Department of Pharmacy, Jiangsu Jianhu People’s Hospital, Yancheng, 224700 China; 2 Department of Pharmacy, First Affiliated Hospital of Nanjing Medical University, No 300 Guangzhou Road, Nanjing City, Jiangsu Province 210029 People’s Republic of China

**Keywords:** Pharmacist intervention, Outpatient prescription, Cost-benefit analysis, Experimental research, Economic evaluation, Drug errors

## Abstract

**Background:**

Limited studies have evaluated the effectiveness of pharmacist interventions on outpatient prescription. The goal of this study was to evaluate the clinical and economic impacts of pharmacist interventions on randomly sampled outpatient prescriptions.

**Method:**

Outpatient prescriptions of our hospital were sampled automatically and reviewed by pharmacists since 2011. Pharmacists intervened in inappropriate prescriptions (IPs) real-timely, and summarized and analyzed the information monthly. Cost-benefit analysis was performed to estimate the economic benefit of the pharmacist intervention.

**Results:**

From 2011 to 2016, pharmacists reviewed 101,271 prescriptions and intervened in 5155 prescriptions. With the interventions of pharmacists, the number of IPs decreased from 1845 to 238, while the inappropriate percentage decreased from 12.60 to 1.22%. The inappropriate rates of different departments and the types decreased annually. IPs were mainly from the Department of Medicine and Department of Surgery and category 1 (Non-indicated medications) in all years. The benefit-to-cost ratios of pharmacist interventions were always more than 1. In the same years, the benefit-to-cost ratios in public payments were higher than those with insurance and self-payment.

**Conclusion:**

This form of pharmacist intervention constitutes a method that showed positive clinical and economic benefits and is worth expanding in large hospitals. Pharmacists should pay more attention on prescriptions in department of surgery or prescriptions with public payments.

## Background

Used appropriately, medications can alleviate distressing symptoms that compromise physical and psychological well-being, help to prevent the onset of many acute and chronic illnesses, and improve patient health outcomes. Too often, however, medications are not used appropriately [1]. In addition to problems involving adverse drug events (ADEs), many patients do not receive optimal pharmaceutical prescriptions. Prescriptions should be reviewed by pharmacists before the medication is administered to the patient in some medical administrative systems, such as the Joint Commission International (JCI) Accreditation Standards for Hospitals [2].

Prescription reviewing is only one aspect of pharmacist intervention. Over the past five decades, pharmacists have attempted to extend their scope of activity beyond the traditional distributive and dispensing roles [3, 4]. Pharmacists will generally intervene in cases of medication problems inclusive of all definitions [5]. Pharmacist interventions were reported to help optimize the process of care by improving the quality of the medication use process and disease management through effective interactions with both patients and other health professionals [6, 7]. However, most of these studies were conducted among inpatient prescriptions.

Pharmacist interventions on outpatient prescriptions were rarely reported. Limited studies were restricted to certain type of outpatient prescriptions, such as prescriptions of pediatric patients [8], geriatric patients [9], emergency patients [10], patients undergoing oral chemotherapy [11, 12] and those with heart failure [13]. These limited articles have all demonstrated that pharmacist intervention can reduce suboptimal prescriptions. However, the effectiveness of pharmacist interventions in overall outpatient prescriptions has not been reported.

One of the best options for outpatient prescriptions intervention is that clinical pharmacists real-timely monitoring all prescriptions and intervening in inappropriate prescriptions (IPs) [14–16]. However, this procedure will not work in China. In our hospital, for example, there are more than 10,000 outpatient prescriptions per day, and each prescription takes a pharmacist 2–3 min to review. If a pharmacist worked 8 h per day, at least 42–63 pharmacists would be needed to review all outpatient prescriptions. Unfortunately, our hospital cannot afford to employ so many pharmacists, and almost none of the hospitals in China can afford to employ the number of pharmacists they would need.

Another method was proposed by the National Health and Family Planning Commission (NHFPC) of the People’s Republic of China in 2010 [17] and has been implemented in more than 100 hospitals since 2011. A certain percentage of prescriptions (more than 1‰ of outpatient prescriptions or more than 1% of inpatient prescriptions) should be sampled randomly and reviewed by pharmacists [17]. Pharmacists collect and categorize IPs and report to doctors and the Hospital Pharmaceutical Administration Committee (HPAC) monthly. Our hospital has implemented this method and has supplemented it with pharmacist real-time intervention since 2011. However, the economic benefits of pharmacist interventions on randomly sampled outpatient prescriptions have not been evaluated. Thus, the goal of this study was to evaluate the clinical effect of pharmacist interventions on outpatient prescriptions, and its distinguishing effect on different departments, inappropriate types and insurance types. We further analyzed the benefit-to-cost ratios of these pharmacist interventions, which indicate the economic effect of pharmacist interventions.

## Methods

### Study design

This study was performed in a Chinese teaching hospital according to an order of the NHFPC [17–19]. The period of current study lasted from January 1, 2011, to December 31, 2016. The outpatient department of this hospital is open 6 days per week and provides services to approximately 10,000 outpatients per day. Outpatient prescriptions were generated via a computerized physician order entry (CPOE) system.

The seventh prescription of every prescriber was automatic sampled by the hospital information system (HIS) every day and sent to the pharmacist workstation. Four experienced pharmacists reviewed these prescriptions. This task was the routine work of the Department of Pharmacy. If a prescription was considered to be potentially IP by pharmacists, the issue was communicated to the prescriber via telephone. If the prescriber accepted the intervention and modified the prescription, the previous prescription was judged as an IP. The information about IPs was recorded on a designated form, based on the standards of the NHFPC [17–19]. The pharmacists completed the forms daily as per the specific categories. All of these jobs took pharmacists four hours per working day. An appointed pharmacist summarized and analyzed the information monthly and sent it to HPAC and the prescribers by office automation system, which required another 16 h per month. The same appointed pharmacist presented and educated about the IP monthly to the medical staff, which required another 2 h per month.

### Description of inappropriate issues

With reference to ASHP guidelines on a standardized method for pharmaceutical care [20], inappropriate issues were divided into 13 categories (Table 1).

**Table 1:** Description and categories of inappropriate issues

Categories	Description
Category 1	Medications with no medical indication
Category 2	Medical conditions for which there is no medication prescribed
Category 3	Medications prescribed inappropriately for a particular medical condition
Category 4	Inappropriate medication dose, dosage form, schedule, route of administration, or method of administration
Category 5	Therapeutic duplication
Category 6	Prescribing of medications to which the patient is allergic
Category 7	Actual and potential clinically significant drug–drug, drug–disease, drug–nutrient, and drug–laboratory test interactions
Category 8	Actual and potential adverse drug events
Category 9	Interference with medical therapy by social or recreational drug use
Category 10	Failure to receive the full benefit of prescribed medication therapy
Category 11	Problems arising from the financial impact of medication therapy on the patient
Category 12	Lack of understanding of the medication therapy by the patient
Category 13	Failure of the patient to adhere to the medication regimen

With reference to ASHP guidelines on a standardized method for pharmaceutical care, [20] inappropriate issues were divided into 13 categories

### Cost-benefit analysis

Cost-benefit analysis was performed to estimate the economic benefits of the pharmacist intervention. Cost was defined as the expenses of pharmacist time [15], which included time for prescription reviewing, intervention, summarizing, analyzing and education. Salary of individual pharmacists is different due to their titles of the professional positions. So we used the average hourly salary of pharmacists, which was calculated based on the annual salary and working hours of a regular pharmacist in our hospital. Other factors such as salary promotion could potentially affect the results. The average hourly salary of a pharmacist was calculated based on the annual salary and working hours of a regular pharmacist in our hospital [15]. Benefit was estimated through cost savings, which was defined as the potentially avoidable cost of inappropriate issues intervened in by pharmacists before the medication was dispensed to the outpatient [15]. Benefit was equal to the sum of the expenses of inappropriate issues. The price of drugs that we used was the price when the prescription was made because it changed three times during our study. All of the costs were recorded in RMB and then converted to US dollars [21]. The final values are reported in US dollars. The cost-benefit analysis is expressed as benefit-to-cost ratios, which were calculated by dividing the cost of IPs by the cost of pharmacist time.

### Statistical analysis

Categorical variables are presented as numbers and percentages, and continuous variables are presented as the means and standard deviations (*SDs*). Continuous variables were tested for normal distribution using the Kolmogorov-Smirnov test and Q-Q plots. Categorical variables were compared using the chi-square test or Fisher’s exact test and continuous variables by the independent sample *t*-test or the Mann-Whitney test. A two-tailed *p* < 0.05 was considered statistically significant. Analyses were performed with SPSS software, version 21 (IBM Corporation, Armonk, New York, USA).

## Results

### General data of the outpatient prescriptions reviewed

The seventh prescription of every prescriber was automatic sampled by the hospital information system (HIS) every day and sent to the pharmacist workstation. Hence, prescriptions from every prescriber in different departments could be sampled. A total of 101,271 outpatient prescriptions were reviewed in this study. The number of reviewed prescriptions increased from 14,646 to 19,567 from 2011to 2016. The percentage of sampling varied from 0.67 to 0.78%. General data of the reviewed prescriptions are shown in Table 2. Approximately one-half of the prescriptions were paid at the patient’s own expense. The number of prescriptions paid by medical insurance increased slowly from 2011 to 2015 but remained less than 50% of the total reviewed prescriptions in 2015.There were no significant changes in age, sex, and expenditure type from 2011 to 2015 (*P* > 0.05).

**Table 2:** General data of reviewed prescriptions

	2011	2012	2013	2014	2015	2016	*P* value
Number of prescriptions	14,646	14,936	15,216	17,961	18,945	19,567	
Percentage of sampling	0.70	0.67	0.68	0.76	0.78	0.78	
Gender							
Male (N, %)	7041 (48.08%)	7122 (47.68%)	7152 (47.00%)	8513 (47.40%)	9121 (48.14%)	9404 (48.06%)	1.000
Female (N, %)	7605 (51.92%)	7814 (52.32%)	8064 (53.00%)	9448 (52.60%)	9824 (51.86%)	10,163 (51.94%)	
Age (Mean ± SD)	52.38 ± 18.85	52.77 ± 19.04	53.75 ± 17.83	52.57 ± 18.92	53.24 ± 19.52	52.74 ± 18.79	0.074
Expenditure type							
Self payment (N, %)	7645 (52.20%)	7833 (52.44%)	8004 (52.60%)	8950 (49.83%)	8881 (46.88%)	8805 (45.00%)	1.000
Public payment (N, %)	1498 (10.23%)	1423 (9.53%)	1412 (9.28%)	1363 (7.59%)	1412 (7.45%)	1454 (7.43%)	
Insurance payment (N, %)	5503 (37.57%)	5680 (38.03%)	5800 (38.12%)	7648 (42.58%)	8652 (45.67%)	9308 (47.57%)	

### Pharmacist intervention reduced the inappropriate percentage gradually

**Fig. 1:**
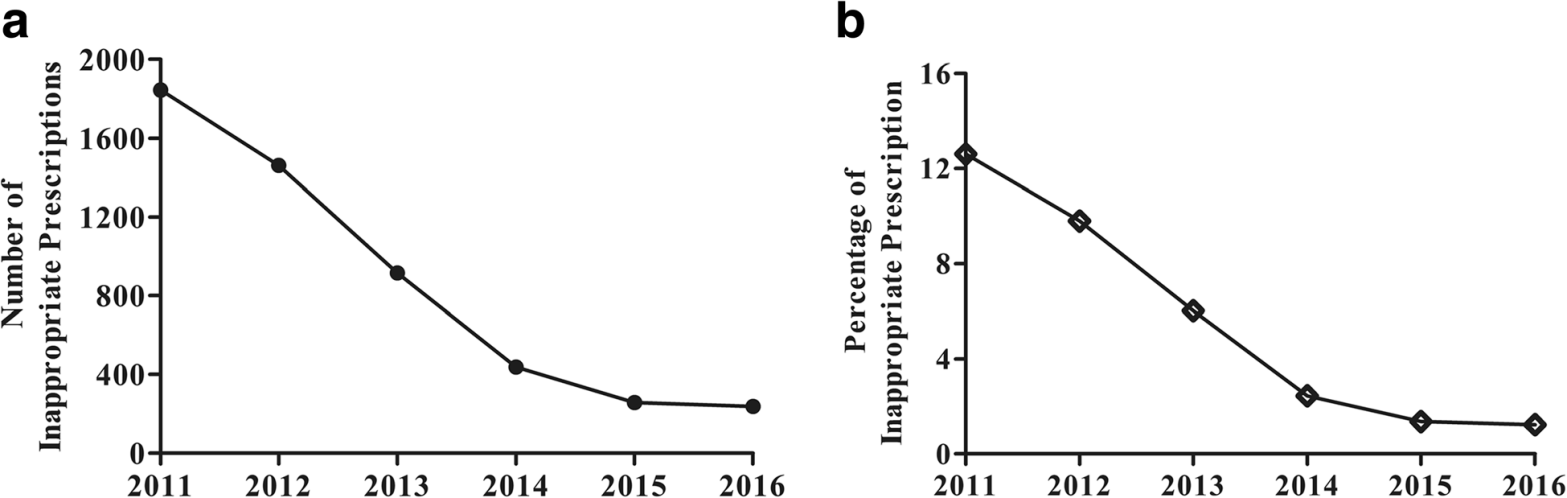
Number (**a**) and percentage (**b**) of inappropriate prescriptions in total

With the intervention of pharmacists, the number and percentage of IPs decreased year by year. The number of IPs decreased from 1845 to 238, while the inappropriate percentage decreased from 12.60 to 1.22% from 2011 to 2016 (Fig. 1). Pharmacist interventions can optimize outpatient prescriptions gradually and continuously.

### Pharmacist intervention reduced IPs in different departments

**Fig. 2:**
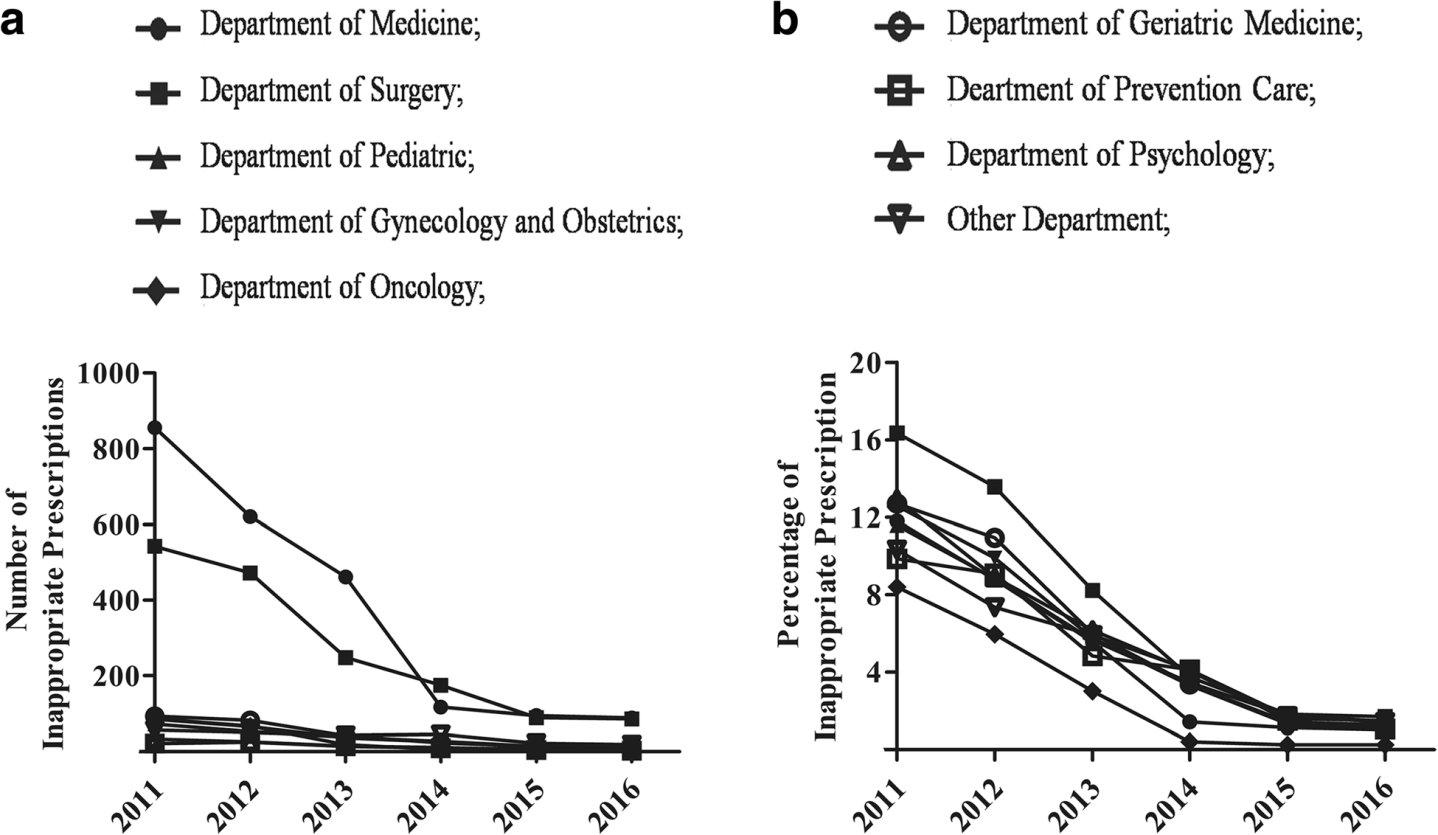
Numbers (**a**) and percentages (**b**) of inappropriate prescriptions in each department

With the intervention of pharmacists, the inappropriate number and percentage in different departments were decreased annually from 2011 to 2016 (Fig. 2). The inappropriate number in the Department of Medicine was decreased from 856 to 92, while that in Department of Surgery decreased from 542 to 89. In 2016, the numbers of IPs in other departments were very few. The inappropriate rate in the Department of Surgery was decreased from 16.34 to 1.70%, while that in Department of Psychology decreased from 12.94 to 1.50%. In 2016, the inappropriate rate in the Department of Surgery and Department of Psychology was higher than that of other departments. The inappropriate percentage in the Department of Oncology was lower than in other departments.

### Pharmacist intervention reduced IPs in different categories

**Fig. 3:**
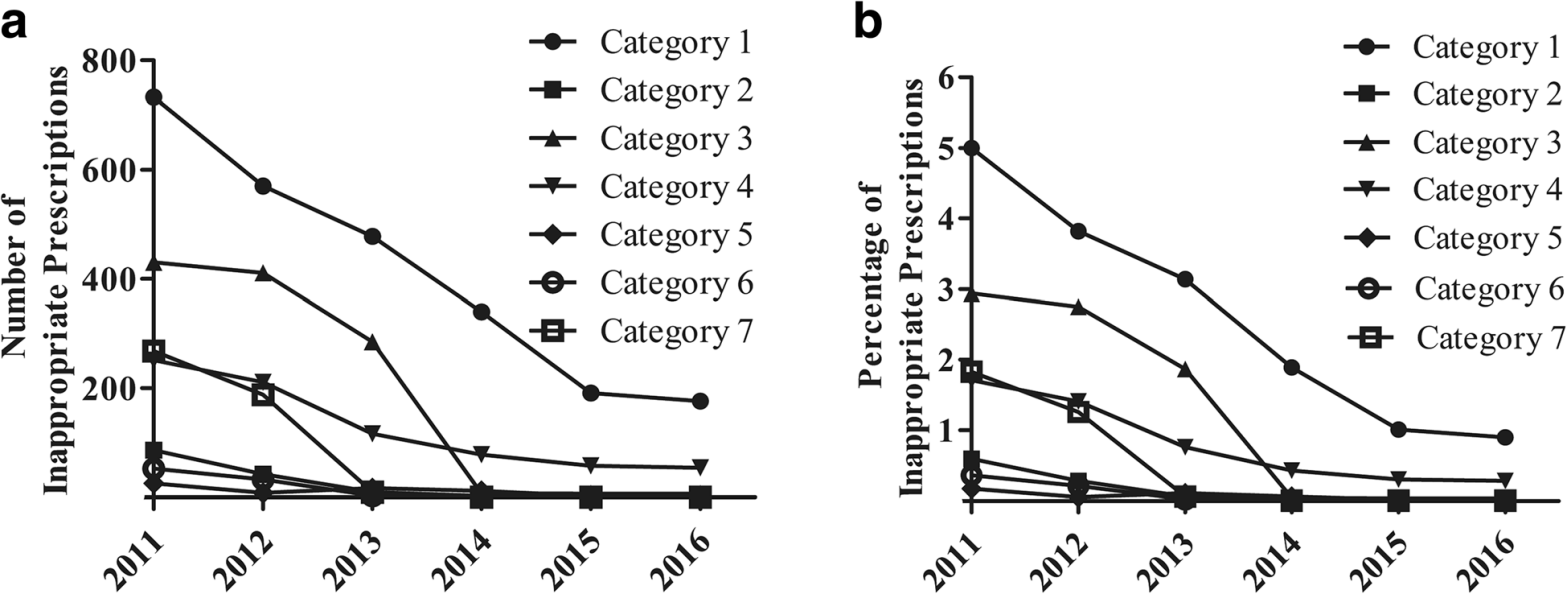
Number (**a**) and percentage (**b**) of each type of inappropriate prescriptions

With the intervention of pharmacists, numbers and percentages of IPs of different types decreased annually in 2011–2016 (Fig. 3). Category 1 (medications with no medical indication) was always the majority inappropriate type, although its percentage decreased annually from 2011 to 2016. Category 3 (inappropriate choice of medication) was the main type of IP in 2011–2013 but was a minority inappropriate type in 2014–2016. Prescriptions of Category 4 (inappropriate medication use) decreased annually in 2011–2016, but constituted 22.69% of IPs in 2016. The proportions of Category 5 (therapeutic duplication) were always low. With the intervention of pharmacists, inappropriate prescriptions of Category 2, 6 and 7 disappeared in 2016. Inappropriate prescriptions of Category 8–13 have not been found in 2011–2016.

### Pharmacist interventions have positive economic effect

From 2011 to 2016, the cost, which defined as the expenses of pharmacist time, grew from $4983.62 to $7867.52. The benefit, which expressed as total cost of all inappropriate issues, decreased from $43,500.30 to $8978.16. The benefit was always higher than the cost, and the benefit-to-cost ratio was always more than 1 (Table 3). This cost-benefit analysis showed positive economic effect for the pharmacist interventions.

**Table 3:** Cost-benefit analysis of prescription intervention by pharmacists

	2011	2012	2013	2014	2015	2016
Hourly salary (Dollar)	4.10	4.66	5.22	5.75	6.31	6.47
Total pharmacist time (Hour) ^a^	1216	1216	1216	1216	1216	1216
Time for reviewing and contacting (Hour) (4 h per working day*250 working days per year)	1000	1000	1000	1000	1000	1000
Time for summarizing and analyzing (Hour) (16 h per month *12 months per year)	192	192	192	192	192	192
Time for education (Hour) (2 h per month *12 months per year)	24	24	24	24	24	24
Total cost of pharmacist time (Dollar)^b^	4984	5666	6352	6987	7673	7868
Total cost of inappropriate prescriptions (Dollar)^c^	43,500	36,718	29,627	15,354	8723	8978
Direct benefit-to-cost ratio ^d^	8.73	6.48	4.66	2.20	1.14	1.14

^a^Total pharmacist time = (Time for reviewing and contacting) + (Time for summarizing and analyzing) + (Time for education);

^b^Total cost of pharmacist time = (Total pharmacist time)* (Hourly salary);

^c^Total cost of inappropriate prescriptions = the sum of costs of all inappropriate items;

^d^Direct benefit-to-cost ratio = (Total cost of inappropriate prescriptions)/(Total cost of pharmacist time);

### Economic effect differs in different payment types

The benefit-to-cost ratio was more than 1 in every payment type in 2011–2016. However, the benefit-to-cost ratio with public payments was higher than those with insurance payment and self-payment in the same year (Table 4). In 2016, the benefit-to-cost with public payments was 1.63, while that that with insurance payment and self-payment was 1.02 and 1.19.

**Table 4:** Cost-benefit analysis of pharmacist interventions in different expenditure types

	2011	2012	2013	2014	2015	2016
Prescription at self payment						
Number of inappropriate prescription	905	720	450	204	116	114
Cost of inappropriate prescription(Dollar)^a^	20,532	17,053	14,103	6730	3844	4212
Cost of pharmacist time(Dollar)^b^	2601	2971	3341	3481	3596	3540
Benefit-to-cost ratio ^c^	7.89	5.74	4.22	1.93	1.07	1.19
Prescription at public payment						
Number of inappropriate prescription	229	171	107	44	28	27
Cost of inappropriate prescription(Dollar)^a^	6624	5701	4231	2086	1001	954
Cost of pharmacist time(Dollar)^b^	510	540	589	530	572	585
Benefit-to-cost ratio ^c^	13.00	10.56	7.18	3.94	1.75	1.63
Prescription at insurance payment						
Number of inappropriate prescription	711	571	359	190	102	97
Cost of inappropriate prescription(Dollar)^a^	16,344	13,964	11,293	6538	3878	3812
Cost of pharmacist time(Dollar)^b^	1873	2155	2422	2976	3505	3743
Benefit-to-cost ratio ^c^	8.73	6.48	4.66	2.20	1.11	1.02

^a^Cost of inappropriate prescription in each expenditure type = the sum of costs of inappropriate items of this type;

^b^We supposed that each prescription took the pharmacist the same time. The time each prescription consumed = (Total pharmacist time)/ (Number of reviewed prescriptions)(see Table 4 and Table 1); Pharmacist time spend on each expenditure type = (The time each prescription consumed)* (Prescription number of this type)(see Table 1); Cost of pharmacist time = (Pharmacist time spend on each expenditure type)* (Hourly salary)(see Table 4);

^c^Benefit-to-cost ratio = (Cost of inappropriate prescription in each expenditure type)/(Total cost of pharmacist time);

## Discussion

Our research revealed that pharmacist interventions could significantly reduced the percentage of irrational outpatient prescriptions, and this randomly sampled pharmacist interventions had positive economic benefits. Our research has implications for clinical practice and future research, particularly with respect to the emerging role that pharmacists have played in rational drug use surveillance in outpatients.

Many studies have shown the efficacy of pharmacist intervention in optimizing inpatient prescribing practices [22–25], but few studies have evaluated the issue in outpatients. Therefore, our study focused on the effectiveness of pharmacist intervention in outpatients. Some advantages of outpatient intervention exist in our hospital. First, the number of outpatients in our hospital was large, confirming that we had a sufficient sample size. Second, the doctors in the outpatient department changed little every year in our hospital. Third, self-payment expenditures were paid to the hospital before the medication was dispensed to the patient. Insurance expenditures and public expenditures were paid by insurance companies and the government on a monthly basis. These advantages made it possible to evaluate the clinical and economic impacts of pharmacist interventions on outpatient prescriptions. In our study, the inappropriate rate of outpatient prescriptions decreased from 12.60 to 1.22% due to pharmacist interventions from 2011 to 2016 (Fig. 1B). The results agreed with those of previous studies in pediatric outpatients [8], geriatric outpatients [9], emergency outpatients [10], outpatients receiving oral chemotherapy [11, 12] and those with heart failure [13].

Randomly sampled prescriptions could well represent the overall inappropriate situation for all prescriptions. It has been reported that the irrational incidence of prescriptions or medication orders was estimated to be 1.59–15.7% with physician-pharmacist team work [24, 26–29]. In our study, the inappropriate rate of sampled prescription was 1.22–12.60% in 2011–2016 (Fig. 1B).

The effectiveness of randomly sampled pharmacist intervention in our study was not much different from that of overall pharmacist intervention. The recently reported incidence of pharmacist intervention in the CPOE system ranged from 0.5 to 4.8% [28–31]. Chappuy M et al.*..* [32] reviewed the outpatient prescriptions of hospital drug sales services in a French university hospital, and 22,279 prescriptions were reviewed over a 1-year period with 247 pharmaceutical interventions (1.1%). Bedouch P et al [24] reported that the incidence of on-ward pharmacist interventions was 15.7% with acceptance of 79.2%. Hence, a rate of 3.26% IPs persisted after the pharmacist intervention. In our study, the inappropriate rate was decreased to as low as 1.22% over the 6-year pharmacist intervention (Fig. 1). The randomly sampled pharmacist intervention had similar effect as the fully sampled or on-ward pharmacist intervention.

Though pharmacist intervention could reduce IPs in all departments, it seemed that pharmacist intervention might be most needed in Department of Surgery. The inappropriate percentage in this department was highest in 2011, and reduced significantly by the pharmacist intervention (Fig. 2). However, the inappropriate percentage in this department was still somewhat higher than other department in 2016. The higher inappropriate percentage may because of the low level of standardization of medication use in this department. There are little guidelines or expert consensuses in surgical disease, and guidelines about medication use in Department of Surgery have not issued in our hospital. Pharmacists should pay more attention on rational use of medication in Department of Surgery. The inappropriate percentage in the Department of Oncology was always lower than the other departments (Fig. 2). There are many recognized guidelines in cancer therapy such as National Comprehensive Cancer Network guidelines, ASCO guidelines, ESMO guidelines CSCO guidelines and so on. According to these guidelines in treatment of disease, the pharmacists had issued a guideline for the rational use of anticancer drugs in 2011 in our hospital. This might an explanation of the lower inappropriate percentage in Department of Oncology.

Though pharmacist intervention could reduce IPs in all categories, it seemed that pharmacist intervention might be most needed in Category 1 (medications with no medical indication). Category 1 (medications with no medical indication) was always the majority inappropriate type, although its percentage decreased annually from 2011 to 2015 (Fig. 3). Another Chinese hospital also reported that 48.54% of urological inpatients undergoing clean or clean-contaminated operations had non-indicated medications before pharmacist interventions [15]. Pharmacists should pay more attention on the irrational issue of medications with no medical indication.

Category 3 (inappropriate choice of medication) decreased rapidly from 2011 to 2016, and became a minority of inappropriate types in 2015. It has reported that most acceptable pharmacist intervention is “inappropriate choices of medications” [24]. As demonstrated by Bedouch P et al. [24], physicians’ acceptance of “inappropriate choices of medications” was 84.1%, which was higher than the average level (79.2%) of pharmacist interventions. This type of irrational issue can improved significantly and quickly by the pharmacist interventions.

Studies of the economic impacts of pharmacist interventions have been limited and have been mostly about inpatients [15, 16, 27, 32–34]. Besides the cost savings, the benefit includes cost avoidance [16] and ultimate improvement. Cost avoidance was defined as the potential economic benefit obtained from interventions that could have prevented ADEs, determined by the total number of intervention cases multiplied by the probability of each potential ADE and the costs associated with ADEs [16]. Ultimate improvement was defined as the practical improvement in health outcome and quality of life. It is very difficult to estimate the cost avoidance and ultimate improvement in outpatients. In our study, benefits included only cost savings, the sum of the expenses for all inappropriate issues. Hence, the benefit of the pharmacist intervention was underestimated in our study.

In our study, the benefit-to-cost ratios of pharmacist interventions were always more than 1, which showed positive economic effect (Table 3). The cost-effective results in our study were not much different from those with reported on-ward pharmacist interventions. Han JM et al.*......* [16] reported that the cost-benefit ratio was 3.64 with pharmacist interventions for large-volume ambulatory-based chemotherapy. Zhang HX et al [15] reported that the cost-benefit ratio was approximately 18.79 with pharmacist interventions for prophylactic antibiotic use in surgical patients undergoing clean or clean-contaminated operations. Ah YM et al [33] reported that the cost-benefit ratio was 3.8 with pharmacist interventions as members of a liver transplant team for hospitalized liver recipients. Rychlíčková J et al [34] reported that the cost-benefit ratio was 3 with clinical pharmacist interventions in the Czech Republic.

The benefit-cost ratio drops from 8.73 in 2011 to 1.14 in 2016, and it shows the great improvement of reduction of IP mainly due to the interventions and education of pharmacists. For example, if levofloxacin was prescripted for the treatment of mammitis, it was judged as IP by the pharmacist, and the issue was communicated to the prescriber via telephone. In the beginning of next month, educations about antibacterial treatment of mammitis were made to all breast surgeons and gynecologists in order to avoid similar IPs. Intervention and education both played an important role in reducing the number and cost of IPs.

Pharmacist intervention was especially important for prescriptions at public payments. The irrational rates and benefit-to-cost ratios of prescriptions at public payments were highest in our study (Table 4). Prescriptions with public payments are totally paid by the government, and they constitute a large proportion of public health expenditures. Prescriptions with insurance payments are supervised by the insurance companies, while prescriptions with public payments aren’t supervised by other organizations. Thus, pharmacist supervision of prescriptions with public payments is especially necessary. There were some limitations of this study. First, this intervention study was performed on the basis of a retrospective design and therefore was less convincing than a prospective, controlled study design. Second, potentially IPs which rejected by doctors were not judged as IPs, which could have led to underestimation of the benefit. Third, the favorable results obtained could not be attributed solely to the pharmacist interventions; therefore, a larger sample size and more rigorous design should be employed to evaluate this promising intervention.

The significance of this study lies in the evaluation of interventions performed routinely over a long period of time by pharmacists in a system in which the role of the pharmacist is stable. This system is the basis for expanding the activities of pharmacists, as well as for the possibility of having their work remunerated by the health care insurance system. Despite its limitations, the economic analysis supported these claims.

## Conclusion

The pharmacists persisted in intervening and reducing the inappropriate percentage of outpatient prescriptions in our hospital from 2011 to 2016. This form of pharmacist intervention constitutes a method that showed positive clinical and economic benefits and is worth expanding in large hospitals. Pharmacists should pay more attention on prescriptions in department of surgery or prescriptions with public.

## Abbreviations

ADEs: Adverse drug events
CPOE: Computerized physician order entry
HIS: Hospital information system.
HPAC: Hospital Pharmaceutical Administration Committee
IP: Inappropriate prescription
NHFPC: National Health and Family Planning Commission
